# PLCG2 signaling and genetic resilience in Alzheimer’s disease

**DOI:** 10.1186/s13024-026-00935-3

**Published:** 2026-03-26

**Authors:** Andy P. Tsai, Amara K. Martin, Andrew Mi, Ava E. Yeh, Eduardo Ramirez Lopez, Tony Wyss-Coray

**Affiliations:** 1 Department of Neurology and Neurological Sciences, Stanford University School of Medicine, Stanford, CA USA; 2 Wu Tsai Neurosciences Institute, Stanford University, Stanford, CA USA; 3 The Phil and Penny Knight Initiative for Brain Resilience, Stanford University, Stanford, CA USA

## Abstract

Alzheimer’s disease (AD) is a progressive neurodegenerative disorder characterized by cognitive decline and pathological hallmarks, including amyloid plaques, tau tangles, microgliosis, and chronic neuroinflammation. Over the past decade, advances in human genetics have revealed microglia and the innate immune pathways are central determinants of AD susceptibility, resilience, and progression, fundamentally redefining the recent conceptual framework of AD research. Genome-wide association studies (GWAS) implicate microglia-enriched genes including triggering receptor expressed on myeloid cells 2 (TREM2), phospholipase-C gamma 2 (PLCG2), and inositol polyphosphate-5-phosphatase D (INPP5D). Among these, the rare PLCG2 P522R variant is associated with reduced AD risk, enhanced microglial responsiveness, and enrichment in cognitively healthy centenarians. Single-cell and spatial transcriptomic studies have uncovered substantial microglial heterogeneity and pronounced region-specific alterations across age and disease progression. These analyses show that microglia transition through a spectrum of transcriptionally distinct states regulated by coordinated remodeling of lipid metabolic, phagocytic and lysosomal pathways, as well as cytokine-receptor signaling networks. Depending on the direction of these state transitions, microglia may engage neuroprotective programs that enhance debris clearance, maintain tissue homeostasis, and support repair, or alternatively, enter maladaptive states characterized by defective lipid processing, chronic inflammatory signaling, and heightened neurotoxicity. Here, we review genetic, molecular, and pharmacological evidence supporting PLCG2 as a compelling therapeutic target in AD. We integrate insights from transcriptomic and structural analyses, iPSC-derived microglia, and in vivo models that show how PLCG2 modulates microglial states, promotes brain resilience, and mitigates AD-related pathophysiology. We also highlight recent progress in identifying small-molecule PLCG2 activators via high-throughput lipid-vesicle assays and affinity-selection mass spectrometry. Collectively, these multidisciplinary advances position PLCG2 as a genetically validated, mechanistically tractable, and pharmacologically actionable target for precision immune-modulation strategies aimed at preserving cognition and enhancing resilience in brain aging and AD.

## Introduction

Alzheimer’s disease (AD) is the most common cause of dementia worldwide [1, 2]. Despite decades of research, effective disease-modifying therapies remain elusive [3]. The global burden of AD is projected to exceed 152 million individuals by 2050, posing an escalating challenge to healthcare systems and society [4].

Aging is the strongest risk factor for AD, yet the development of AD is not an inevitable consequence of aging. Epidemiological, genetic, and biological studies reveal that some individuals possess resilience factors that alter their disease susceptibility and reshape the trajectories of AD risk [5–7]. These observations have shifted attention toward mechanisms that promote resilience rather than solely those that drive pathology.

Immune pathways have emerged as central determinants of AD risk and progression. Recent genome-wide association studies (GWAS) have identified over 80 genetic loci associated with AD risk, with approximately half of these susceptibility genes linked to immune-related pathways [8]. Intriguingly, rare variants have been associated with reduced AD risk [9–11], including PLCG2 P522R variant (PLCG2^P522R^; rs72824905). PLCG2 is expressed across multiple immune cell types, including microglia within the central nervous system as well as peripheral myeloid cells and lymphoid cells [12–14]. While protective effects associated with PLCG2 variants may reflect coordinated actions across immune compartments, converging genetic, expression, and functional evidence indicates that microglia are likely key mediators of PLCG2-dependent effects in brain aging and AD [14–16].

Microglia, the brain’s resident immune cells, play essential roles in maintaining brain homeostasis including synaptic pruning, debris clearance and phagocytosis; however, chronic or dysregulated activation can lead to neuroinflammation, lipid droplet accumulation, and synaptic dysfunction [17–20]. Rather than broad immune suppression, selective enhancement of protective microglial pathways may offer therapeutic benefits. Understanding how genetic resilience against AD shapes microglial states may inform the development of precision immune-modulation strategies.

In this review, we consolidate current insights into PLCG2 biology by integrating human genetic evidence with molecular, cellular, and pharmacological studies. We examine how PLCG2 signaling regulates immune responses relevant to brain aging and AD, with a particular focus on microglia as candidate mediators of central nervous system effects. We also highlight emerging therapeutic strategies that seek to recapitulate genetically defined resilience mechanisms, while discussing key challenges, including sex-specific immune responses, the TREM2-PLCG2 signaling axis, translational barriers, and the need for personalized immune-modulatory approaches across diverse populations.

### Human genetic evidence for resilience and disease risk

AD is a complex disorder regulated by both risk and resilience factors across a diverse polygenic landscape [8]. While variants such as Apolipoprotein E (APOE) ε4 and TREM2 R47H (TREM2^R47H^) are associated with increased AD risk [21, 22], growing attention has shifted toward alleles linked to reduced disease susceptibility that may point to mechanisms of resilience [23]. APOE ε2, for example, is associated with reduced AD risk and amyloid burden, and has also been linked to increased longevity [24]. However, rare homozygosity for APOE ε2 is associated with hyperlipoproteinemia and increased risk of premature atherosclerosis [25, 26].

More recently, *PLCG2* has emerged as a compelling gene in human studies. The rare missense variant PLCG2^P522R^ (allele frequency ~ 0.67%) represents one of the most consistently validated genetic resilience modifiers of AD risk, identified across multiple GWAS and associated with a significant reduction in disease risk (odds ratios ranging from approximately 0.63 to 0.76) [10–28]. Beyond disease susceptibility, PLCG2^P522R^ is enriched among cognitively healthy centenarians and has been associated with enhanced brain resilience and a 1.49-fold increased likelihood of longevity [29, 30].

Notably, other PLCG2 variants are associated with either increased AD risk [31] or with immune dysregulation diseases such as PLCG2-associated antibody deficiency and immune dysregulation (PLAID) and autoinflammatory PLAID (APLAID) [32, 33]. Together, these observations highlight the critical importance of signaling balance, as both insufficient and excessive PLCG2 activity can drive maladaptive immune responses, whereas finely tuned modulation appears necessary to promote immune resilience and neuroprotection.

Clinical studies further support the functional relevance of the PLCG2^P522R^ to human disease trajectories. In a longitudinal cohort of 3,595 individuals with mild cognitive impairment (MCI), carriers of PLCG2^P522R^ exhibited significantly slower cognitive decline and lower cerebrospinal fluid (CSF) levels of phosphorylated tau (p-tau181) compared to non-carriers [34]. Statistical mediation analyses indicated that reduced tau pathology accounted for a substantial proportion of the cognitive benefit, with an effect size comparable in magnitude, but opposite in direction, to that of APOE ε4 [34]. Notably, the protective effect of PLCG2^P522R^ was most pronounced in MCI individuals with established amyloid pathology, suggesting that this variant may modulate disease progression downstream of amyloid pathology, thereby mitigating tau accumulation and cognitive deterioration. Supporting the broader relevance of these protective effects, rare coding variants in PLCG2, alongside other immune-related genes such as TREM2 and ABI family member 3 (ABI3), have been identified in admixed population, including Argentinian cohorts, indicating that PLCG2-mediated resilience is not restricted to European ancestry [35]. Together, these human genetic and clinical observations position PLCG2 as a modifier of AD risk and disease progression, reinforcing brain resilience across diverse genetic backgrounds. Finally, PLCG2 functions within a broader AD genetic network that includes APOE, TREM2, and INPP5D, supporting the concept that resilience arises from coordinated modulation of immune pathways rather than single-gene effects.

### PLCG2 molecular function, signaling pathways, and variant-specific effects

PLCG2 is predominantly expressed in cells of the innate immune system across both mice and humans, including myeloid cells, macrophages, NK cells, B cells, and microglia, with comparatively low expression in non-immune tissues [12, 13]. Transcriptomic analyses from bulk and single-cell datasets showed that PLCG2 is enriched within immune-associated transcriptional programs across peripheral organs and the central nervous system [14, 15]. Within the brain, PLCG2 expression is restricted to microglia, where transcript levels are elevated in plaque-associated microglia [36]. Importantly, transcript abundance does not always correspond to protein levels or enzymatic activity, and systematic analyses of PLCG2 protein expression or in vivo enzymatic activity across tissues and disease states remain limited. Accordingly, current insights into PLCG2 function are derived primarily from genetic, transcriptomic, and functional perturbation studies, with relatively few direct measurements of PLCG2 enzyme activity in physiological or disease contexts.

**Fig. 1:**
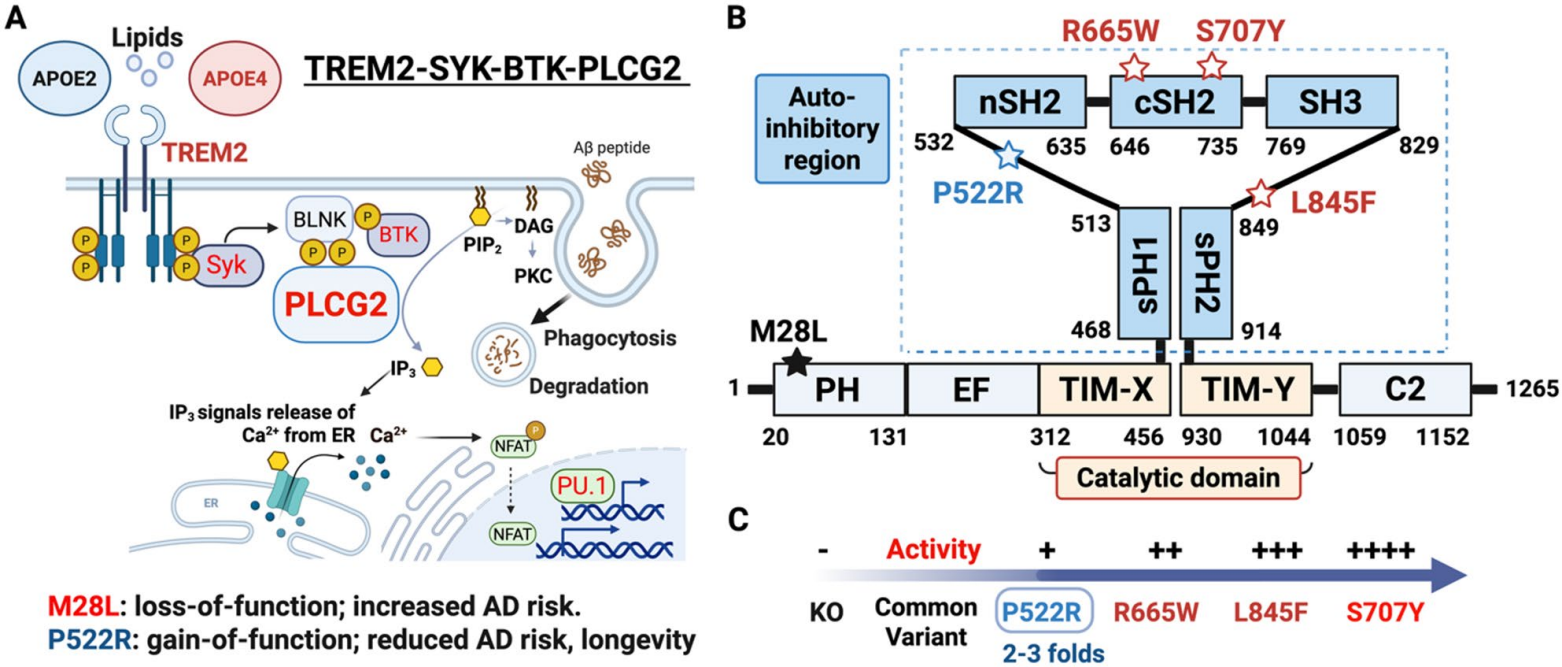
TREM2-SYK-BTK-PLCG2 signaling pathway and activity of PLCG2 variants. (**A**) Schematic illustrating how lipids and lipid-associated proteins (e.g., APOE2, APOE4) engage the microglial receptor TREM2, leading to phosphorylation of SYK and downstream activation of the BLNK-BTK-PLCG2 signaling axis. Activated PLCG2 hydrolyzes PIP₂ into IP₃ and DAG, triggering intracellular Ca²⁺ release, PKC activation, and induction of transcriptional programs such as NFAT and PU.1. These pathways coordinate microglial effector functions, including phagocytosis, lipid degradation, and responses to Aβ peptide. (**B**) PLCG2 domain architecture highlighting the autoinhibitory region and catalytic domain, with protective and disease-associated variants mapped by structural position. (**C**) PLCG2 variants are arranged according to their relative enzymatic activity. Loss-of-function variants such as M28L increase AD risk, whereas the protective variant P522R confers a mild gain-of-function (2-3-fold increased activity) associated with reduced AD risk and enhanced likelihood of longevity. Strong hypermorphic mutations (R665W, L845F, S707Y) exhibit progressively higher PLCG2 activity

PLCG2 functions as a canonical downstream effector of immunoreceptor signaling. Upon ligand binding, TREM2 interacts with the adaptor protein TYROBP (also known as DAP12), initiating phosphorylation cascades involving spleen tyrosine kinase (SYK) [37, 38] and Bruton’s tyrosine kinase (BTK) [39]. BTK phosphorylates PLCG2 (potential tyrosine residues; Y759 and Y1217), leading to its activation [40]. Activated PLCG2 hydrolyzes phosphatidylinositol 4,5-bisphosphate (PIP₂) into two critical second messengers: diacylglycerol (DAG) and inositol 1,4,5-trisphosphate (IP₃). This triggers intracellular calcium release and protein kinase C (PKC) activation, driving downstream transcriptional and metabolic responses [13, 15]. However, it remains unclear whether PLCG2 activation always requires phosphorylation (Fig. 1A).

Genetic variants in PLCG2 provide important insight into how distinct modes of enzyme modulation shape microglial function. AD- and longevity-associated PLCG2^P522R^ resides between an atypical split pleckstrin homology domain and the C-terminus Src homology 2 domain and induces a modest gain-of-function effect, resulting in approximately a 2-3-fold increase in enzymatic activity [15]. This subtle enhancement is thought to promote moderately increased innate immune signaling without overt immune dysregulation (Fig. 1B).

In contrast, the missense M28L variant (PLCG2^M28L^; rs61749044; allele frequency: 1.48%), which maintains normal basal activity following Rac2 GTPase stimulation [41], is associated with increased AD risk [15, 31]. Notably, carriers of PLCG2^M28L^ are resistant to BTK inhibitors [33, 42]. Given that BTK inhibition reduces phagocytosis and suppresses synaptic structure uptake in rodent microglia, these observations further implicate the BTK-PLCG2 signaling axis as a critical regulator of microglial function [43].

A further contrast is illustrated by the hypermorphic PLCG2 mutations. The hypermorphic R655W (~ 20-fold) and L845F (~ 61-fold) mutations have been identified in chronic lymphocytic leukemia (CLL) with BTK inhibitor (Ibrutinib) resistance, while the S707Y (~ 120-fold) mutation is associated with both CLL and autoinflammatory syndromes PLAID and APLAID (Fig. 1C; Table 1) [32, 44–46]. Despite markedly increased PLCG2 enzymatic activity, the S707Y mutation is associated with impaired microglial functions, including reduced phagocytosis and cytokine production [47]. Transcriptomic profiling of S707Y-expressing microglia reveals downregulation of genes involved in complement signaling, phagocytosis, and interferon responses, consistent with a dysfunctional and desensitized immune state [47].

Structural and functional studies reveal that PLCG2^P522R^ modestly enhances PLCG2 activity by disrupting autoinhibitory interactions, enabling more efficient, receptor-dependent engagement with membrane phosphoinositides [48, 49]. This finely tuned activation supports the notion that mild, context-specific potentiation, rather than broad hyperactivation, might be sufficient for therapeutic benefit. Accordingly, pharmacologic strategies that replicate the activation profile of PLCG2^P522R^ may optimize microglial function while avoiding immune overactivation or exhaustion, offering a balanced approach to AD intervention [49].

Together, these contrasting outcomes underscore the importance of precise modulation of PLCG2 activity, rather than indiscriminate enhancement, for maintaining effective microglial immune function in neurodegenerative disease (Fig. 1).

**Table 1:** Variant-specific effects of PLCG2 on enzymatic activity and disease association

Variant	Allele Frequency	Activity	AD	Ibrutinib resistance	PLAID / APLAID	CLL
M28L	1.48E-02	-	V	V	X	X
P522R	6.74E-03	~2-3-fold	V	X	X	X
R665W	1.24E-06	~20-fold	X	V	X	V
S707Y	Somatic; CLL	~120-fold	X	V	V	V
L845F	Somatic; CLL	~60-fold	X	V	X	V

### PLCG2 expression across tissues, aging, and disease

Given the high expression of PLCG2 in the hematopoietic system, Magno et al. examined PLCG2 distribution in human and mouse brain tissue using immunohistochemistry and in situ hybridization [13]. In the adult mouse brain, PLCG2 was found largely restricted to microglia, with limited expression observed in granule cells [13]. Pharmacological depletion of microglia using colony-stimulating factor 1 receptor (CSF1R) inhibitors leads to diminished brain PLCG2 expression, whereas microglial repopulation restores it [14]. In the context of brain aging, distinct regions exhibit age-associated transcriptional changes, and recent work has shown that *Plcg2* displays a unique age-related transcriptional pattern in several regions, including the posterior hippocampus [50]. However, this region-specific regulation will require validation at single-cell resolution.

In AD-affected brain regions, PLCG2 expression correlates strongly with amyloid burden, but not tau pathology, suggesting there may be a preferential association with Aβ-driven pathology [14]. However, the role of PLCG2 in tau-related pathology remains unclear, as it has not been systematically examined in either in vivo or in vitro tau models. Microglia with high PLCG2 expression exhibit elevated stress-responsive signatures, including unfolded protein response and NF-κB signaling [14].

Importantly, PLCG2 expression in the brain differed between 5xFAD mice (amyloidosis models) harboring the PLCG2^M28L^ and PLCG2^P522R^ variants. Consistent with these transcriptional differences, PLCG2 protein levels were reduced in the brains of 5xFAD; M28L mice (0.47-fold relative to 5xFAD mice), with a significant difference observed between 5xFAD; M28L and 5xFAD; P522R mice. Reduced protein expression was also detected in the spleens of 5xFAD; M28L mice, although the mechanisms underlying this reduction remain unknown [15].

Together, there are currently no comprehensive studies systematically assessing PLCG2 protein abundance across species and tissues. Future studies are therefore needed to elucidate how PLCG2 protein expression, variant-specific regulation, and enzymatic activity are coordinated across tissues during aging and disease.

### In vivo modeling of PLCG2 and disease-associated variants

PLCG2 is highly expressed in plaque-associated microglia, particularly those encircling dense-core Aβ deposits [13, 14]. These microglia exhibit transcriptional programs enriched in lipid metabolism, lysosomal biogenesis, and antigen presentation, pathways that are partially regulated by PLCG2. In 5xFAD mice, PLCG2 expression increases with disease progression and correlates with microglial clustering around plaques, underscoring its association with microgliosis and amyloid burden [14].

Functionally, PLCG2 is critical for microglial-plaque interactions. In 5xFAD mice, *Plcg2* haploinsufficiency, accompanied by reduced TREM2 expression, exacerbates amyloid pathology by impairing microglial clustering and phagocytic gene expression, resulting in larger, less compact plaques [51]. Conversely, the PLCG2^P522R^ variant enhances Aβ uptake and promotes dense-core plaque formation, whereas the PLCG2^M28L^ loss-of-function variant disrupts these processes [15]. The SYK-PLCG2 signaling also drives the plaque-associated PU.1 (encoded by the *SPI1* gene) low microglial state, as genetic deletion of either SYK or PLCG2 reduces microglial engagement with plaques and attenuates the prevalence of PU.1 low expression microglia [52]. Together, these findings establish PLCG2 as a key mediator of amyloid containment and plaque-associated microglial states, highlighting its potential as a therapeutic target.

Beyond amyloid pathology, PLCG2 is also involved in regulating inflammatory responses within the brain [53]. Loss-of-function leads to impaired interleukin (IL)-10 production and altered levels of TNF-α, IL-6, and IL-4, skewing the immune response toward a maladaptive pro-inflammatory state. In contrast, the PLCG2^P522R^ variant appears to fine-tune this response, enhancing clearance mechanisms while avoiding excessive inflammation, an effect that may underlie its protective association in AD [15, 54]. Transcriptomic profiling further supports this interpretation, as PLCG2^P522R^-expressing microglia show upregulation of genes involved in phagocytosis and immune modulation (*Axl*, *Cd9*, *Csf1r*, *Itgax*, *Hif1a*), lipid metabolism (*Apoe*, *Lgals3*), and oxidative stress responses (*Cybb*, *Lilr4b*, *Tmem163*). These signatures define two neuroprotective microglial states, activated plaque-responsive (Act) A and B, enriched for endocytic, migratory, and apoptotic pathways. Compared with wild-type or PLCG2^M28L^ risk variant microglia, PLCG2^P522R^ microglia preferentially adopt these protective states linked to efficient clearance and tissue repair without excessive inflammation [15].

**Fig. 2:**
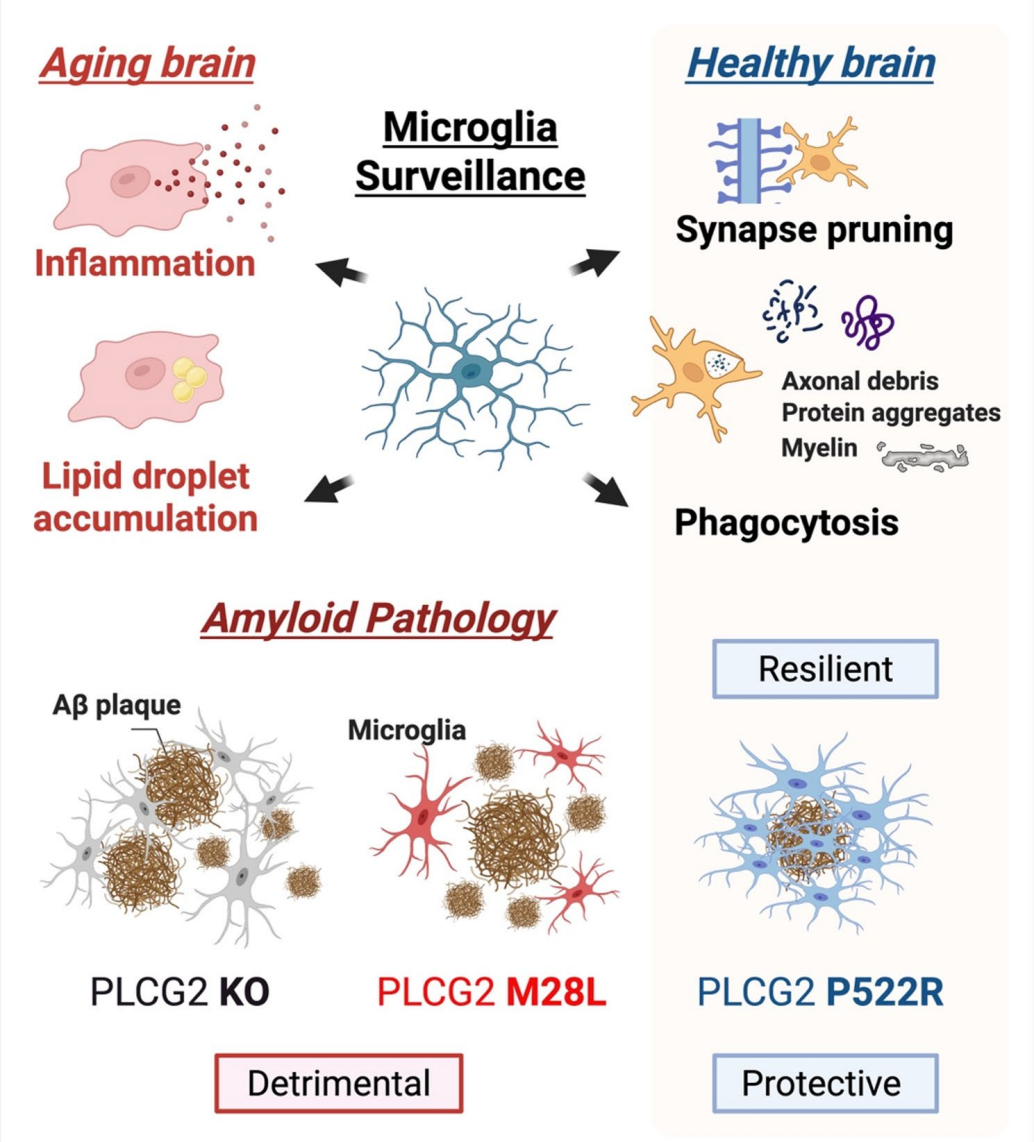
Microglial surveillance and PLCG2-dependent modulation of brain resilience in aging and AD. Microglia maintain tissue homeostasis through surveillance, synapse pruning, and phagocytosis in the healthy brain. Aging impairs these functions, leading to inflammation and lipid-droplet accumulation. In amyloid pathology, PLCG2 deficiency or the AD-risk variant PLCG2^M28L^ results in detrimental microglial responses. In contrast, the AD-protective variant PLCG2^P522R^ enhances microglial engagement with Aβ plaques and promotes resilient, neuroprotective states

PLCG2 signaling also influences synaptic integrity [55]. While microglial phagocytosis is essential for debris clearance, excessive pruning can be detrimental to neuronal function. In 5xFAD mice, PLCG2^M28L^ accelerates synapse loss and cognitive decline, whereas PLCG2^P522R^ carriers exhibit preserved synaptic plasticity and improved neuronal function. These findings suggest that PLCG2 contributes to synaptic resilience by calibrating microglial activation thresholds [15, 56], thereby balancing clearance functions, lipid metabolism, and inflammatory responses with synaptic preservation (Fig. 2).

Although most in vivo studies have focused on amyloid pathology, emerging human data suggest a potential link between PLCG2 and tau-driven neurodegeneration. The protective PLCG2^P522R^ variant is associated with slower cognitive decline, lower CSF tau and p-tau181 in MCI patients, and mitigated tau pathology in neuropathologically confirmed dementia with Lewy bodies (DLB) and progressive supranuclear palsy (PSP) cases [29, 57]. These observations raise the possibility that PLCG2 modulates tau-related neurodegenerative processes, although in vivo tauopathy mouse models directly interrogating this mechanism have not yet been examined.

Beyond population-level associations, in vivo chimeric models provide additional insight into how PLCG2^P522R^ confers resilience. In chimeric AD mice generated by transplanting human iPSC-derived microglia carrying PLCG2^P522R^, enhanced microglial antigen presentation and chemokine signaling are observed, accompanied by increased recruitment of CD8-positive T cells to the brain and activation of transcriptional programs associated with immune surveillance [54]. Emerging evidence suggests that adaptive immune cells, including CD8⁺ and CD4⁺ T cells, contribute to AD progression. Clonally expanded T cells have been identified in AD brain tissue and cerebrospinal fluid, and alterations in T-cell receptor repertoires have been associated with disease severity [58]. In addition, recent studies indicate that T-cell-microglia interactions may influence neuroinflammatory response and neuronal vulnerability [59]. Together, these findings suggest that PLCG2^P522R^ not only slows neurodegeneration but also modulates protective immune interactions that may limit disease progression. However, the mechanisms by which PLCG2^P522R^ drives CD8-positive T-cell infiltration into the brain, as well as the roles these T cells play within the brain, remain unclear.

Furthermore, recent in vivo work demonstrates that PLCG2 variants may act as risk modifiers in a context-dependent manner. A study by the IU/JAX/PITT MODEL-AD consortium introduced the PLCG2^M28L^ risk variant [15, 31, 60] into a mouse model carrying APOE ε4 and TREM2^R47H^ (LOAD1). When challenged with a high-fat/high-sugar diet, LOAD1; PLCG2^M28L^ mice exhibited accelerated AD-relevant phenotypes, including increased cortical microgliosis, altered brain metabolism, and transcriptomic signatures that closely mirrored human AD pathology [60, 61]. These effects were sex-specific and diet-dependent, with female mice showing stronger microglial activation and region-specific metabolic alterations, while males displayed distinct peripheral cytokine responses [60]. These observations highlight the importance of gene-environment interactions in PLCG2 function and underscore the need to consider sex as a biological variable in preclinical studies of PLCG2-targeted interventions in the animal models. However, the sex-dependent effects are still unclear in human studies.

Collectively, in vivo studies indicate that the PLCG2^P522R^ variant fine-tunes microglial function by promoting a disease-associated microglia (DAM)-like phenotype while avoiding excessive inflammation, suggesting a favorable recalibration of immune signaling that enhances resilience [15, 62]. Together, genetic, clinical, and mechanistic evidence highlights PLCG2 as a key regulator of microglial resilience and a compelling therapeutic target. In contrast to non-specific anti-inflammatory therapies, which have largely failed in AD clinical trials, precision targeting of PLCG2 may offer a disease-contextual strategy to enhance neuroprotective immune states in the aging and AD brain.

### In vitro modeling and mechanistic dissection

PLCG2 deficiency has been associated with transcriptional changes in pathways related to TREM2-dependent calcium signaling, cytoskeleton dynamics, and phagocytic function [51]. Functionally, PLCG2 knockout abolishes TREM2-evoked calcium flux/inositol monophosphate accumulation and reduces cytoskeleton-dependent adhesion and phagocytic activity in human iPSC-derived myeloid cells [63]. Consistent with these findings, iPSC-derived microglia lacking PLCG2 exhibit defective uptake of myelin debris, diminished clearance of Aβ aggregates, and reduced expression of phagocytic receptors [64].

Beyond phagocytosis, PLCG2 regulates lipid metabolic pathways that are critical for microglial survival, energy homeostasis, and activation [65, 66]. PLCG2-deficient iPSC-derived microglia show reduced expression of genes involved in lipid uptake and storage, including *LPL*, *APOC1*, *FABP5*, and *PLIN2*, leading to impaired lysosomal function and compromised responses to pathological stress [64, 67]. Supporting these cellular observations, lipidomic analyses revealed that PLCG2 depletion in young mouse brains is associated with substantial reductions in myelin-specific lipid species, accompanied by transcriptomic alterations in multiple myelin-related genes [67]. Together, these findings link PLCG2 signaling to lipid-lysosomal integrity and myelin homeostasis.

Mechanistically, co-expression network analyses have placed PLCG2 within a functional module that includes APOE and TREM2, enriched for complement cascade genes and microglial markers characteristic of DAM state [14, 51, 68]. Studies in human iPSC-derived microglia show that TREM2 signals through PLCG2 to regulate survival, phagocytosis, and lipid metabolism, whereas TREM2-independent PLCG2 signaling downstream of Toll-like receptors mediates inflammatory responses, positioning PLCG2 as a central integrator of microglial state transitions during neurodegeneration [64].

To identify potential PLCG2 activators, a lipid vesicle-based fluorogenic assay (XY-69) has been developed that reconstitutes PLCG2-mediated hydrolysis of PIP2 within a membrane-mimetic environment, enabling fluorescence-based quantification of enzymatic activity [69, 70]. This high-throughput screening (HTS) platform enabled the evaluation of ~ 6,000 compounds for their ability to enhance cellular membrane-dependent PLCG2 activity [49], providing a powerful tool for mechanistic and pharmacological interrogation.

From the screen, flupirtine (AC1) emerged as a promising lead compound, which was on the clinical trials for multiple sclerosis to prevent neurodegeneration beyond inflammation [71, 72]. Flupirtine binds PLCG2 with moderate affinity (the dissociation constant, KD, ~ 20 µM), enhances enzymatic activity in vitro, and promotes microglial phagocytosis in cellular assays, without inducing excessive cytokine release [49, 71]. Notably, this activation profile resembles the modest gain-of-function conferred by the protective PLCG2^P522R^, suggesting that flupirtine may potentiate microglial function while avoiding inflammatory overstimulation.

Consistent with this interpretation, flupirtine has demonstrated neuroprotective effects in several in vivo models. In experimental autoimmune encephalomyelitis (EAE), a model of multiple sclerosis, it preserved retinal ganglion cell integrity and visual function, reduced axonal damage and demyelination, without affecting systemic immune responses [73]. Similarly, in a chronic restraint stress model, flupirtine rescued spatial memory deficits, reduced hippocampal apoptosis, and preserved synaptic integrity, changes accompanied by activation of pro-survival pathways such as Akt, GSK-3β, and Erk1/2 [74]. Whether these neuroprotective actions are directly mediated by PLCG2 activation remains unclear.

Complementary efforts using affinity-selection mass spectrometry could identify additional PLCG2-binding small molecules. These candidates can then be evaluated through orthogonal validation approaches, including cellular thermal shift assays to assess direct target engagement and protein stabilization, enzymatic assays to quantify PLCG2 catalytic activity, and high-content imaging to evaluate functional outcomes such as phagocytosis and inflammatory signaling. Initial screening may be performed in established microglial cell lines, but validation in more physiologically relevant systems, such as human iPSC-derived microglia or primary microglial cultures, will be essential to confirm translational relevance. Compounds demonstrating favorable pharmacodynamic properties may further be assessed for blood-brain barrier permeability and central nervous system (CNS) drug-likeness, supporting their consideration for future in vivo studies.

Subsequent medicinal chemistry efforts subsequently focused on refining molecular scaffolds to enhance PLCG2 membrane-dependent activation while minimizing off-target effects on related isoforms such as PLCG1 [49]. A key priority was preserving the context-dependent activation profile of PLCG2, avoiding compounds that induce constitutive activation or disrupt autoinhibitory domains in a non-selective manner. This strategy aims to recapitulate the nuanced, membrane-specific modulation achieved by the protective PLCG2^P522R^ variant, thereby maximizing therapeutic efficacy while reducing safety risks.

### Microglia as candidate, but not exclusive, mediators

Converging genetic, transcriptomic, and functional evidence position microglia as primary mediators of PLCG2-dependent effects within the central nervous system. In vivo and in vitro studies consistently demonstrate that modulation of PLCG2 activity shapes microglial responses to amyloid pathology, synaptic remodeling, and neurodegenerative stress, supporting a central role for microglia in translating PLCG2 generic variation into brain-relevant resilience phenotypes.

PLCG2 is broadly expressed across peripheral immune cell types such as B cells, macrophages, and dendritic cells, indicating that PLCG2-associated protective effects may involve coordinated contributions from both CNS-resident and peripheral immune compartments [12, 40, 75]. Consistent with this possibility, carriers of the AD-protective PLCG2^P522R^ variant (aged 59–103 years) exhibit a slightly more responsive immune system, with increased PLCG2 phosphorylation and calcium flux in B cells upon B-cell receptor stimulation, as well as enhanced myeloid reactive oxygen species production during Fc-receptor-mediated phagocytosis of opsonized E. coli [76]. These considerations argue that the most viable PLCG2-directed strategies should aim for CNS-preferential and microglia-biased modulation, ideally recapitulating the modest gain-of-function profile of PLCG2^P522R^ rather than inducing broad hyperactivation.

PLCG2-targeted therapies may synergize with other emerging immune-modulating strategies for AD, including TREM2 agonists, BTK inhibitors, CSF1R inhibitors, and SYK-targeted interventions [18, 43, 52]. Combination approaches that recalibrate multiple nodes of the microglial activation network may offer superior efficacy compared to monotherapies. Specifically, PLCG2 activation may enhance the efficacy of anti-amyloid immunotherapies by boosting microglial capacity for plaque clearance and mitigating treatment-associated inflammation. Early integration of PLCG2 modulators into combinatorial therapeutic designs holds promise for amplifying neuroprotective microglial phenotypes, although such combinatorial effects remain to be tested.

### Therapeutic perspective

Despite strong genetic and preclinical evidence supporting PLCG2 as a therapeutic target, several critical challenges must be addressed to realize its clinical potential. Precision modulation of innate immune pathways in the brain, while promising, demands a cautious and nuanced approach to balance efficacy and safety. One of the foremost challenges is achieving selective modulation of PLCG2 activity within the CNS while minimizing peripheral immune perturbations, where dysregulated activation can promote autoimmunity or inflammatory disorders [12, 44].

The protective PLCG2^P522R^ variant induces a modest gain-of-function, suggesting that only partial activation of PLCG2 is beneficial [49]. Excessive activation may phenocopy hyperactivating PLCG2 mutations associated with immune dysfunction or neurotoxicity. Defining the therapeutic window for PLCG2 activation is therefore paramount. Dose-finding studies in preclinical models must evaluate not only amyloid clearance but also effects on synaptic integrity, inflammatory tone, and overall cognitive outcomes.

An additional barrier is effective delivery of PLCG2-targeted agents to microglia across the blood-brain barrier, as many small molecules with in vitro activity fail to achieve sufficient CNS exposure. In this context, robust biomarkers of PLCG2 engagement and downstream signaling, including phosphorylation status, downstream calcium signaling, and lipid metabolic gene programs, will be essential to guide dose optimization and de-risk early-phase clinical development.

## Conclusion

Over the past decade, evidence has established PLCG2 as a critical regulator of microglial function and a promising therapeutic target for AD [14, 15]. The protective PLCG2^P522R^ variant offers a naturally occurring example of how subtle enhancement of microglial immune competence can confer resilience against neurodegeneration without inducing detrimental inflammation. Through its central role in the TREM2-SYK-BTK signaling axis, PLCG2 coordinates essential microglial functions that maintain brain homeostasis, including amyloid plaques and apoptotic debris clearance, lipid metabolism, inflammatory responses, and preservation of synaptic integrity [64]. Loss of PLCG2 function exacerbates amyloid pathology and accelerates cognitive decline, while controlled activation, as exemplified by PLCG2^P522R^, promotes neuroprotective microglial states [15].

Therapeutic strategies have recently focused on recapitulating the beneficial effects of PLCG2^P522R^ through small-molecule activation of PLCG2. High-throughput screening platforms such as the XY-69 lipid vesicle assay and affinity selection mass spectrometry have identified promising lead compounds with CNS-penetrant properties and selective activation profiles [49].

Precision medicine approaches, incorporating genetic background, sex, and aging factors, will be key to maximizing the benefits of PLCG2-targeted interventions. Importantly, the potential impact of PLCG2 modulation may extend beyond AD. Enhancing microglial resilience through targeted PLCG2 activation could offer therapeutic opportunities in other neurodegenerative diseases and even contribute to promoting cognitive longevity during normal aging.

While the primary focus of PLCG2 modulation has been in AD, emerging evidence indicates broader relevance across brain aging and diverse neurodegenerative or inflammatory conditions. PLCG2 is implicated in multiple sclerosis [77], Parkinson’s disease [12], and systemic autoimmune disorders [32], suggesting that targeted fine-tuning of innate immunity may offer therapeutic benefit beyond AD.

Future research should extend beyond amyloid-centered mechanisms to define how PLCG2 contributes to additional dimensions of brain resilience, including synaptic plasticity, neuronal survival, and cognitive longevity. The intersection of PLCG2 biology with aging processes opens intriguing possibilities for preventive interventions aimed at maintaining brain health across the lifespan.

In summary, PLCG2 exemplifies how insights from human genetic resilience can inform therapeutic development. By leveraging endogenous protective or resilience mechanisms, PLCG2-targeted treatments may re-define how we prevent, delay, and treat AD. Continued scientific leadership has been critical for advancing this progress by enabling open-access tools, stringent target validation, and comprehensive preclinical platforms. These collaborative frameworks are paving the way toward a new era of precision neuroimmunology and healthy brain aging.

## Data Availability

No datasets were generated or analysed during the current study.

## Abbreviations

AD: Alzheimer’s disease
GWAS: Genome-wide association studies
TREM2: Triggering receptor expressed on myeloid cells 2
PLCG2: Phospholipase-C gamma 2
INPP5D: Inositol polyphosphate-5-phosphatase D
APOE: Apolipoprotein E
CLU: Clusterin
ABI3: ABI family member 3
Aβ: Amyloid-β
MCI: Mild cognitive impairment
CSF: Cerebrospinal fluid
DAM: Disease-associated microglia
BTK: Bruton’s tyrosine kinase
LOAD1: Late-onset AD 1
TYROBP: TYRO protein tyrosine kinase-binding protein
SYK: Spleen tyrosine kinase
PIP₂: Phosphatidylinositol 4,5-bisphosphate
DAG: Diacylglycerol
IP₃: Inositol 1,4,5-trisphosphate
PKC: Protein kinase C
LPL: Lipoprotein lipase
APOC1: Apolipoprotein C1
FABP5: Fatty acid-binding protein 5
PLIN2: Perilipin 2
APLAID: Autoinflammation-PLCG2-associated antibody deficiency and immune dysregulation
DLB: Dementia with Lewy bodies
PSP: Progressive supranuclear palsy
NF-κB: Nuclear Factor kappa-light-chain-enhancer of activated B cells
CSF1R: Colony-Stimulating Factor 1 Receptor
IL: Interleukin
TNF-α: Tumor necrosis factor-alpha
HTS: High-throughput screening
EAE: Experimental autoimmune encephalomyelitis
CNS: Central nervous system
PET: Positron emission tomography
